# Exploring the role of non-coding RNAs as potential candidate biomarkers in the cross-talk between diabetes mellitus and Alzheimer’s disease

**DOI:** 10.3389/fnagi.2022.955461

**Published:** 2022-08-24

**Authors:** Shokoofeh Ghiam, Changiz Eslahchi, Koorosh Shahpasand, Mehran Habibi-Rezaei, Sajjad Gharaghani

**Affiliations:** ^1^Laboratory of Bioinformatics and Drug Design, Institute of Biochemistry and Biophysics, University of Tehran, Tehran, Iran; ^2^Department of Computer and Data Sciences, Faculty of Mathematical Sciences, Shahid-Beheshti University, Tehran, Iran; ^3^School of Biological Sciences, Institute for Research in Fundamental Sciences (IPM), Tehran, Iran; ^4^Department of Stem Cells and Developmental Biology, Cell Science Research Center, Royan Institute for Stem Cell Biology and Technology (RI-SCBT), Tehran, Iran; ^5^Department of Cell and Molecular Biology, School of Biology, College of Science, University of Tehran, Tehran, Iran

**Keywords:** biomarker detection, Alzheimer’s disease (AD), diabetes mellitus (DM), type 3 diabetes mellitus, brain diabetes, genome wide association study, non-coding RNA, bioinformatics

## Abstract

**Background:**

Recent research has investigated the connection between Diabetes Mellitus (DM) and Alzheimer’s Disease (AD). Insulin resistance plays a crucial role in this interaction. Studies have focused on dysregulated proteins to disrupt this connection. Non-coding RNAs (ncRNAs), on the other hand, play an important role in the development of many diseases. They encode the majority of the human genome and regulate gene expression through a variety of mechanisms. Consequently, identifying significant ncRNAs and utilizing them as biomarkers could facilitate the early detection of this cross-talk. On the other hand, computational-based methods may help to understand the possible relationships between different molecules and conduct future wet laboratory experiments.

**Materials and methods:**

In this study, we retrieved Genome-Wide Association Study (GWAS, 2008) results from the United Kingdom Biobank database using the keywords “Alzheimer’s” and “Diabetes Mellitus.” After excluding low confidence variants, statistical analysis was performed, and adjusted *p*-values were determined. Using the Linkage Disequilibrium method, 127 significant shared Single Nucleotide Polymorphism (SNP) were chosen and the SNP-SNP interaction network was built. From this network, dense subgraphs were extracted as signatures. By mapping each signature to the reference genome, genes associated with the selected SNPs were retrieved. Then, protein-microRNA (miRNA) and miRNA-long non-coding RNA (lncRNA) bipartite networks were built and significant ncRNAs were extracted. After the validation process, by applying the scoring function, the final protein-miRNA-lncRNA tripartite network was constructed, and significant miRNAs and lncRNAs were identified.

**Results:**

Hsa-miR-199a-5p, hsa-miR-199b-5p, hsa-miR-423-5p, and hsa-miR-3184-5p, the four most significant miRNAs, as well as NEAT1, XIST, and KCNQ1OT1, the three most important lncRNAs, and their interacting proteins in the final tripartite network, have been proposed as new candidate biomarkers in the cross-talk between DM and AD. The literature review also validates the obtained ncRNAs. In addition, miRNA/lncRNA pairs; hsa-miR-124-3p/KCNQ1OT1, hsa-miR-124-3p/NEAT1, and hsa-miR-124-3p/XIST, all expressed in the brain, and their interacting proteins in our final network are suggested for future research investigation.

**Conclusion:**

This study identified 127 shared SNPs, 7 proteins, 15 miRNAs, and 11 lncRNAs involved in the cross-talk between DM and AD. Different network analysis and scoring function suggested the most significant miRNAs and lncRNAs as potential candidate biomarkers for wet laboratory experiments. Considering these candidate biomarkers may help in the early detection of DM and AD co-occurrence.

## Introduction

Diabetes Mellitus (DM) and Alzheimer’s Disease (AD) are two of the most prevalent diseases in the elderly population of the world. DM cases are increasing exponentially, and by 2035 there will likely be 592 million cases. Moreover, the incidence of DM-induced AD in the human population has increased rapidly over the past decade. DM not only increases the risk of AD, but also doubles or triples the development rate of it. Given the apparent relationship between these two diseases, it has been suggested that AD is actually a neuroendocrine disorder and not just a neurologic one. Similar to insulin resistance in the brain, AD brains have decreased glucose uptake from the environment. Patients with DM and AD have amyloid beta deposits in the pancreas and brain. In addition, certain DM target receptors are involved in the regulation of tau expression and phosphorylation, a significant target protein in AD. This phenomenon is referred to as the cross-talk between DM and AD (Kandimalla et al., 2017).

Diabetes Mellitus and its complications are primarily caused by insulin resistance and impaired insulin signaling. Insulin encourages cells to absorb glucose. It is also required for normal cognitive function of hippocampus. Several studies have found that brain insulin resistance is a key factor in the link between DM and AD (Baglietto-Vargas et al., 2016; Spinelli et al., 2019; McNay and Pearson-Leary, 2020). There are additional cellular and molecular reasons besides impaired insulin signaling. The majority of the main evidence includes oxidative-stress, inflammation, obesity, dysregulation of Apolipoprotein E (APOE), insulin degradation enzyme (IDE), Glucose Transporter type 4 (GLUT4), and Acetylcholinesterase (AChE), are linked to insulin resistance (Mittal and Katare, 2016).

Mittal et al. (2016) derived differentially expressed proteins of Type 2 Diabetes Mellitus (T2DM) and AD individually 70 proteins in total and retrieved the first neighbor of each protein in protein-protein interaction (PPI) network (957 proteins). Using pathway analysis and interactions between these proteins, the most relevant proteins for both T2DM and AD were identified (Mittal et al., 2016). Karki et al. (2017) used Biological Expression Language (BEL) to identify six significant shared pathways between T2DM and AD at both the molecular and clinical levels. They also discovered that some drugs used to treat T2DM might increase the risk of AD (Karki et al., 2017). Another research (Shakil, 2017) demonstrated that antidiabetic drugs can be used to treat Alzheimer’s disease. Based on this hypothesis, molecular docking was used to study two antidiabetic drugs, ertugliflozin and sotagliflozin, and their targets, sodium glucose cotransporter 2 and AChE, respectively. Finally, sotagliflozin structure was proposed for designing drugs that may treat DM and AD simultaneously. Hu et al. (2020) retrieved data from the ROSMAP Project for T2DM and AD, analyzed multi-omic data using an inference method and identified common pathways between these two diseases.

Furthermore, variations in the human genome may increase the risk of chronic diseases like DM and AD. As a result, Genome-Wide Association Study (GWAS) projects were conducted to identify new loci linked to both diseases. Hao et al. (2015) investigated shared genetic risk factors in the association between T2DM and AD using a systems biology approach. They discovered genes involved in both diseases using GWAS analysis. By combining gene ontologies, co-expression networks, and regulatory elements, they discovered shared Single Nucleotide Polymorphisms (SNPs) and their associated genes as novel targets for further investigation. In order to identify the most important pathways in the cross-talk between T2DM and AD, they finally used pathway enrichment analysis.

Despite the importance of protein dysregulation in the development of many diseases, recent research has revealed that non-coding RNAs (ncRNAs) also play a significant role in the progression of complex diseases (Li and Kowdley, 2012; Natarajan et al., 2013; DiStefano, 2018; Lekka and Hall, 2018). Furthermore, the majority of the human genome encodes ncRNAs, with only a small percentage converting to proteins. Therefore, researchers believe that ncRNAs have a significant impact on neurodegeneration processes that lead to AD (Idda et al., 2018) and metabolic disorders that lead to DM (Alvarez and Distefano, 2013). Approximately 70% of experimentally detected microRNAs (miRNAs) are expressed in the brain and are involved in memory and synapses formation (Silvestro et al., 2019). Additionally, they regulate the function of insulin and, consequently, their dysregulation has been linked to the progression of DM (Nigi et al., 2018). On the other hand, long non-coding RNAs (lncRNAs), also have been linked to insulin resistance, inflammation, and DM (Sathishkumar et al., 2018), as well as the progression of AD (Luo and Chen, 2016).

Despite producing some promising and valuable insights, less attention has been paid to the impact of ncRNAs on the cross-talk between DM and AD to date. Therefore, this research is motivated by the fact that, while dysregulated proteins play an important role in the progression of many diseases, ncRNAs also have a strong impact on complex diseases. Consequently, using GWAS results and statistical and network analysis, we proposed candidate miRNAs and lncRNAs as potential biomarkers for early detection of the co-occurrence of DM and AD. The following covers data collection, networks analysis, validations, and scoring function in the section “Materials and methods.” Significant ncRNAs in the final tripartite network were introduced in the section “Result.” We believe that the paths in this network from lncRNAs to proteins (which pass through miRNAs) recommend potential candidate biomarkers that may also be drug targets in the cross-talk between DM and AD. We will discuss them in detail in the section “Discussion.”

## Materials and methods

### Data set

We retrieved GWAS results using keywords “Alzheimer’s” and “Diabetes” from Neale Lab^1^, which performed GWAS analysis from the United Kingdom Biobank database (Bycroft et al., 2018). For DM and AD 35 and 11 files were downloaded in both sexes, respectively. GWAS results in each file included genotyped and imputed data on 13,791,467 SNPs from more than 300,000 volunteers in both diseases. The beta coefficient, *p*-value, minor allele frequency, and standard error were all included in each dataset. To find protein-miRNA and miRNA-lncRNA interactions, we used the Starbase (2010) database (Li et al., 2014).

### Mapping single nucleotide polymorphisms to proteins

Initially, significant SNPs from each disease should be chosen. We employed two statistical parameters: Minor Allele Frequency (MAF) and adj.*p*-value. MAF is a standard used to determine the frequency of the second most common allele for a particular SNP. Due to the fact that variants with low confidence (MAF < 0.001) did not meet the MAF threshold, they were removed from the DM and AD datasets. The adj.*p*-value for the remaining SNPs was calculated using the FDR method in R. Significant SNPs were determined to have adj.*p*-value < 0.05. Following data cleaning, significant SNPs revealed by both diseases were selected.

LD is a commonly used method for calculating SNP correlation. It was employed to identify interactions between SNPs. It is only applicable to SNPs located on the same chromosome. It generates two disequilibrium values; D’ and *R*^2^, describing how frequently alleles are inherited together. LD performed a chi-squared test for each pair of SNPs and calculates D’ (S_*i*_, S_*j*_) and *R*^2^ (S_*i*_, S_*j*_) and reports the *p*-value (S_*i*_, S_*j*_) (Slatkin, 2008). We calculated LD using the NIH API in R (National Cancer Institute^2^). Then, we created the graph G_*s*_ which its nodes represent the significant SNPs that were identified in the preceding step. We considered an edge between two SNPs if D’ (S_*i*_, S_*j*_) >0.7 and *p*-value (S_*i*_, S_*j*_) < 0.0001. This graph contains of four connected components. Two components correspond to SNPs on chromosome 19, and the other two components correspond to SNPs on chromosome 6. For each component, we looked for dense subgraphs that contained at least 20% of the nodes in that component. Then, the nodes of each dense subgraph are regarded as a collection of SNP’s signatures.

To identify genes (proteins and ncRNAs) associated with the signatures, we utilized the UCSC Genome Browser^3^ and retrieved information about the genes and their biological consequences. SNPnexus^4^ was then used to annotate the achieved genes and their corresponding proteins. Next, the Disgenet database (2014), a database for disease gene associations, was used to determine if these proteins are linked to both DM and AD.

### Protein-miRNA and miRNA-long non-coding RNA networks

Assume that p_1_, p_2_, …, p_*n*_ are proteins achieved from the previous step and m_1_, m_2_, …, m_*l*_ are miRNAs, obtained from the starbase database, that interacts with at least one of these proteins. The protein-miRNA bipartite network was subsequently created. The nodes of one part are the set *P* = {p_1_, p_2_, …, p_*n*_} and the other part are the set *M* = {m_1_, m_2_, …, m_*l*_}. If miRNA m_*j*_ regulates protein p_*i*_, there is an edge between p_*i*_ and m_*j*_. The top 10 miRNAs with the highest degree in the corresponding network were selected as significant, formally denoted by *M*’ = {m’_1_, m’_2_, m’_3_,., m’_*s*_}. Next, suppose that l_1_, l_2_, …, l_*r*_ are lncRNAs, retrieved from the starbase database, that interact with at least one of the miRNAs m’_*i*_. Similarly, the miRNA-lncRNA bipartite network was constructed. The nodes of one part is the set M’ and the other part is the set *L* = {l_1_, l_2_, …, l_*r*_}. There is an edge between m’_*i*_ and l_*j*_ if lncRNA l_*j*_ regulates miRNA m’_*i.*_ In the constructed network, similarly to previous network, the top 10 lncRNAs with the highest degree were chosen as significant, formally denoted by *L* ‘ = {l’_1_, l’_2_, l’_3_,., l’_*t*_}.

### Protein-miRNA-long non-coding RNA tripartite network

After passing validation, the final protein-miRNA-lncRNA tripartite network was constructed. The first layer of this network is represented by the set P, the second by the set M’, and the third by the set L’. Similar to the previous bipartite networks, if miRNA m’_*j*_ regulates protein p_*i*_, there is an edge between p_*i*_ and m’_*j*_. In addition, there is an edge between m’_*i*_ and l’_*j*_ if lncRNA l’_*j*_ regulates the miRNA m’_*i.*_ To validate the edges of this network we considered the following well-known databases. We used TargetScan (2005), miRwalk (2011), microT (2012), and miRmap (2012) databases to validate protein-miRNA interactions and LncBase (2012) database to validate miRNA-lncRNA interactions. If an edge was reported in at least one of the aforementioned databases, the interaction was kept in the final network; otherwise, it was removed.

### Scoring function

By assigning a score to ncRNAs, the significance of the nodes in the tripartite network was determined. Assume that *N*_*P*_ is the total number of proteins in the final tripartite network and NP(m)′i denotes the set of proteins regulated by miRNA m′i. The scoring function was calculated as follows:


(1) S⁢(m′i)=NP(m)′iNP

(2) S⁢(l′i)=∑m′jϵNm′(l′i))S⁢(m′j)

Where Nm′⁢(l′i) denote the set of miRNAs which regulated by lncRNA l′i.

In fact, to calculate S⁢(l′i), all the miRNAs in the tripartite network, regulated by lncRNA l′i were retrieved and the sum of the scores of the corresponding miRNAs was assigned as the score of the intended lncRNA.

**FIGURE 1:**
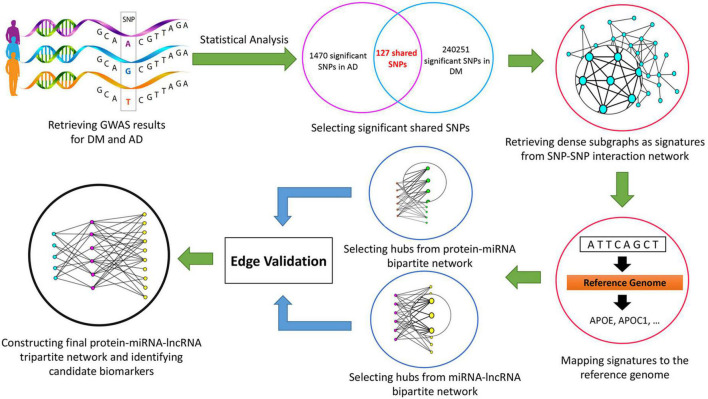
The workflow of the study.

The research workflow is depicted in Figure 1.

## Results

### Networks analysis

**FIGURE 2:**
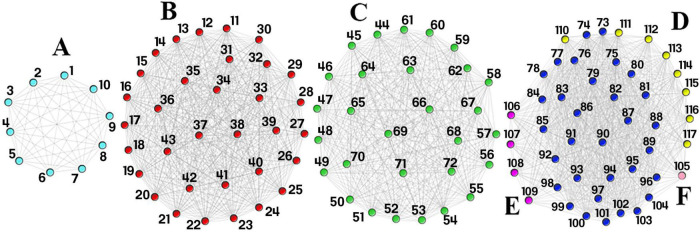
Single Nucleotide Polymorphism (SNP)-SNP interaction network. This network consists of four components. Component 1 consists of signature (A) (light blue nodes) and component 2 consists of signature (B) (red nodes). Component 3 consists of signature (C) (green nodes) and component 4 consist of signatures (D–F). The dark blue nodes in the last component are shared by signatures (D–F) while the yellow, purple, and light pink nodes are unique to signatures (D–F), respectively. The first two components are found on chromosome 19, while the last two are found on chromosome 6. Each SNP rsid number is listed in Table 1.

There were 127 common SNPs among the 1470 and 240251 significant SNPs identified by analyzing AD and DM GWAS results, respectively. Forty-three SNPs occurred on chromosome 19 and 84 SNPs on chromosome 6. Supplementary Table 1 contains all the information about common SNPs revealed by both diseases. To construct the SNP-SNP interaction network LD was calculated and different D’ value thresholds were tested. The final network consists of four components. Two components are associated to chromosome 19, while the remaining two are linked to chromosome 6. As we have already mentioned, to identify signatures, we considered dense subgraphs containing at least 20% of the chromosome’s total nodes. As a result, the subgraphs linked to chromosomes 19 should contain at least 8 and subgraphs linked to chromosomes 6 should contain at least 16 nodes. Each of the 43 SNPs on chromosome 19 belongs to at least one signature. Ten of the 84 SNPs on chromosome 6 do not participate in any signature; some are members of small dense subgraphs (which do not meet the signature threshold), while others do not form any dense subgraph. The results are shown in Table 1 and Figure 2.

**TABLE 1:** Common significant SNPs between DM and AD.

1	rs112588918	21	rs157581	41	rs157592	61	rs112077259	81	rs35743245	101	rs34331363
2	rs2199575	22	rs34095326	42	rs111789331	62	rs2894188	82	rs36096565	102	rs35226637
3	rs57537848	23	rs34404554	43	rs66626994	63	rs3095242	83	rs35972518	103	rs35653258
4	rs11666329	24	rs11556505	44	rs576224725	64	rs3095241	84	rs35917796	104	rs2647059
5	rs2972559	25	rs157582	45	rs3095250	65	rs3130413	85	rs35380574	105	rs2647062
6	rs71338733	26	rs59007384	46	rs3132496	66	rs2394906	86	rs35395738	106	rs558721
7	rs73050205	27	rs769449	47	rs3095248	67	rs3130416	87	rs35472547	107	rs679242
8	rs199956232	28	rs429358	48	rs3130712	68	rs3130418	88	rs34939562	108	rs2760990
9	rs4803763	29	rs75627662	49	rs3130406	69	rs3134750	89	rs34924558	109	rs2647066
10	rs4803764	30	rs10414043	50	rs3130535	70	rs3130431	90	rs34496598	110	rs17425622
11	rs142042446	31	rs7256200	51	rs3130688	71	rs9264187	91	rs35525122	111	rs601148
12	rs12972156	32	rs483082	52	rs3134766	72	rs7769393	92	rs34350244	112	rs601945
13	rs12972970	33	rs438811	53	rs3130536	73	rs4458721	93	rs34535888	113	rs3130411
14	rs34342646	34	rs34954997	54	rs3134764	74	rs35899943	94	rs34553045	114	rs9271494
15	rs283811	35	rs5117	55	rs3134763	75	rs1980496	95	rs2760980	115	rs6917729
16	rs283815	36	rs12721046	56	rs2394900	76	rs9268433	96	rs2760983	116	rs6605556
17	rs6857	37	rs12721051	57	rs34763471	77	rs3793127	97	rs113134061	117	rs9268455
18	rs71352238	38	rs56131196	58	rs2394901	78	rs3763309	98	rs2760984		
19	rs184017	39	rs4420638	59	rs3095244	79	rs3763312	99	rs2454139		
20	rs2075650	40	rs814573	60	rs3134757	80	rs9269041	100	rs34117221		

The SNPs represented in this table are depicted in Figure 2.

**FIGURE 3:**
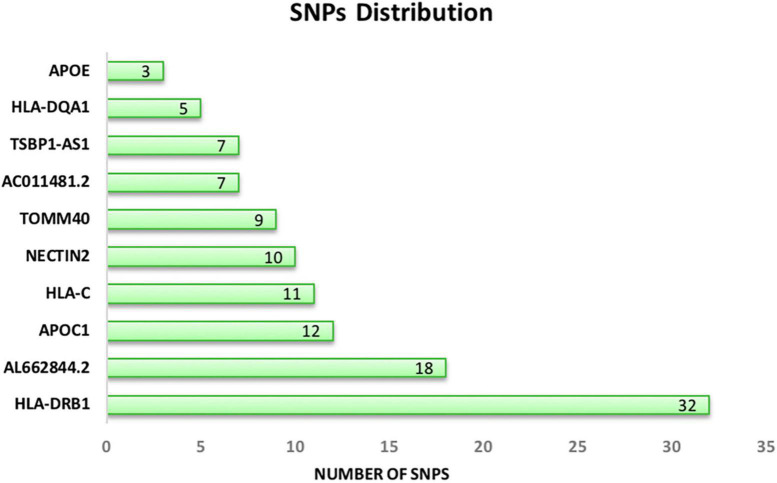
Distribution of common significant SNPs on the associated genes. APOE (chromosome 19) and HLA-DRB1 (chromosome 6) have the lowest and the highest number of SNPs.

After mapping each signature to the reference genome, 7 proteins, 2 lncRNAs, and one pseudogene were identified. Genes with at least 3 SNPs were reported in our dataset. According to Figure 3, HLA-DRB1and APOE have the highest and lowest number of SNPs, respectively. Signatures and their associated genes are displayed in Table 2.

**TABLE 2:** Mapping signatures to the reference genome.

**Gene**	**Annotation**	**Chr No.**	**Signature ID**
NECTIN2	Protein coding	19	A
AC011481.2	lncRNA	19	B
TOMM40	Protein coding	19	B
APOE	Protein coding	19	B
APOC1	Protein coding	19	B
AL662844.2	Unprocessed pseudogene	6	C
HLA-C	Protein coding	6	C
HLA-DRB1	Protein coding	6	D, E
HLA-DQA1	Protein coding	6	D, E, F
TSBP1-AS1	lncRNA	6	D, F

This table contains the annotation of the obtained genes, the chromosome number, and the corresponding signature. Some SNPs are found in multiple signatures.

To highlight the importance of miRNAs in the progression of both diseases we created a protein-miRNA bipartite network. This network showed that among 238 miRNAs, hsa-miR-199a-5p, hsa-miR-199b-5p, hsa-miR-423-5p, hsa-miR-3184-5p, hsa-miR-124-3p, hsa-miR-506-3p, hsa-miR-1321, hsa-miR-4731, hsa-miR-491-5p, hsa-miR-663a, hsa-miR-744-5p, hsa-miR-665, hsa-miR-1286, hsa-miR-1908-5p, and hsa-miR-873-5p are the top 10 high-degree miRNAs in the network. Since some nodes shared the same degree, 15 miRNAs were chosen.

Next, miRNA-lncRNA bipartite network was built and high degree lncRNAs were chosen. Among 350 lncRNAs in this network, Nuclear paraspeckle assembly transcript 1 (NEAT1), AC069281.2, X inactive specific transcript (XIST), KCNQ1 opposite strand/antisense transcript 1 (KCNQ1OT1), AC010442.1, AC092127.1, SLC9A3-AS1, KRTAP5-AS1, AC010327.5, STAG3L5P-PVRIG2P-PILRB, and LINC00963 are the top 10 high-degree ones. Since some nodes shared the same degree, 11 lncRNAs were chosen. Supplementary Tables 2, 3 contain all the protein-miRNA and miRNA-lncRNA interactions.

### Validations

To ensure that the achieved proteins are associated with the mentioned diseases, we used the Disgenet database. The findings revealed that APOE is a key gene in the progression of both diseases. APOC1, TOMM40, and NECTIN2 play a bigger role in AD than in DM, while HLA-C, HLA-DQA1, and HLA-DRB1 play a bigger role in DM than in AD. Nevertheless, all proteins are involved in both diseases. Results have been shown in Table 3.

**TABLE 3:** Protein validation.

Protein	Disease	N_PMIDs
APOE	Alzheimer’s disease	3042
	Alzheimer’s disease, late onset	431
	Diabetes mellitus	87
	Diabetes mellitus, non-insulin-dependent	83
	Diabetes mellitus, insulin-dependent	14
APOC1	Alzheimer’s disease	20
	Alzheimer’s disease, late onset	5
	Diabetic nephropathy	1
TOMM40	Alzheimer’s disease	92
	Alzheimer’s disease, late onset	24
	Diabetes mellitus, non-insulin-dependent	4
NECTIN2	Alzheimer’s disease	25
	Alzheimer’s disease, late onset	6
	Diabetes mellitus, non-insulin-dependent	1
HLA-C	Alzheimer’s disease	2
	Diabetes mellitus, insulin-dependent	24
HLA-DQA1	Alzheimer’s disease	1
	Diabetes mellitus, insulin-dependent	191
	Diabetes mellitus, non-insulin-dependent	3
HLA-DRB1	Alzheimer’s disease	10
	Alzheimer’s disease, late onset	1
	Diabetes mellitus, insulin-dependent	279
	Diabetes mellitus, non-insulin-dependent	17

To increase confidence to the final network, all interactions were double-checked against TargetScan miRwalk, miRmap, microT and LncBase databases. As shown in Table 4, all protein-miRNA interactions passed the validation process and no edge has been removed. However, according to Table 5, some edges did not satisfy the validation process for miRNA-lncRNA interactions and were removed.

**TABLE 4:** Protein-miRNA interaction validation.

	APOC1	APOE	HLA-C	HLA-DQA1	HLA-DRB1	TOMM40	NECTIN2
miR-199a-5p	**a, b**	0	0	**a, b**	0	**a, b, c**	**a, b**
miR-199b-5p	**a, d**	0	0	**a, f**	0	**a, b, c**	**a, b**
miR-423-5p	0	0	**a, b**	**a, b**	0	**a, b**	**a, b**
miR-3184-5p	0	0	**a, b**	**a, b**	0	**a, b**	**a, b**
miR-124-3p	**a, d**	0	0	**a, b**	0	**a, e**	0
miR-506-3p	**a, b**	0	0	**a, b**	0	**a, b**	0
miR-1321	**a, b**	0	0	0	**a, b, c**	**a, b**	0
miR-4731-5p	**a, b, c**	0	0	0	0	**a, b**	**a, b**
miR-491-5p	0	**a, e**	**a, b, c**	0	0	**a, b**	0
miR-663a	0	**a, b, c, e**	0	0	0	**a, b**	**a, b**
miR-744-5p	0	**a, b**	0	0	0	**a, b**	**a, b**
miR-665	0	**a, b, c**	0	0	0	**a, b**	**a, b**
miR-1286	0	**a, e**	0	0	**a, b**	**a, e**	0
miR-1908-5p	0	**a, b, c, e**	0	0	0	**a, b**	**a, b**
miR-873-5p	0	0	**a, c**	0	0	**a, b**	**a, c**

In this table, all protein-miRNA interactions were validated with other databases. In the table, a: Starbase database, b: miRwalk database, c: TargetScan database, d: miRanda database (accessed from starbase database), e: miRmap database, f: microT database. “0” denotes there is no interaction between intended protein and miRNA. Edges that passed the validation process has been bolded.

**TABLE 5:** MiRNA-lncRNA interaction validation.

	NEAT1	AC069281.2	XIST	KCNQ1OT1	AC010442.1	AC092127.1	SLC9A3-AS1	KRTAP5-AS1	AC010327.5	STAG3L5P	LINC00963
miR-124-3p	**S, L**	S	**S, L**	**S, L**	**S, L**	0	0	0	S	0	0
miR-1286	S	S	0	0	0	S	0	0	S	0	0
miR-1321	**S, L**	**S, L**	**S, L**	**S, L**	S	S	S	S	0	S	S
miR-1908-5p	S	S	S	0	0	S	S	S	0	0	S
miR-199a-5p	0	0	0	**S, L**	0	0	0	**S, L**	0	S	S
miR-199b-5p	0	0	0	**S, L**	0	0	0	**S, L**	0	S	S
miR-3184-5p	**S, L**	S	**S, L**	**S, L**	S	**S, L**	S	S	S	S	0
miR-423-5p	**S, L**	S	**S, L**	**S, L**	**S, L**	**S, L**	**S, L**	**S, L**	**S, L**	**S, L**	0
miR-4731-5p	**S, L**	**S, L**	**S, L**	**S, L**	0	S	S	S	S	0	**S, L**
miR-491-5p	**S, L**	S	**S, L**	**S, L**	**S, L**	0	0	0	0	S	0
miR-506-3p	**S, L**	S	**S, L**	**S, L**	**S, L**	0	0	0	S	0	0
miR-663a	S	S	S	0	0	S	S	S	0	0	S
miR-665	**S, L**	S	**S, L**	**S, L**	0	0	S	0	S	S	**S, L**
miR-744-5p	0	0	0	0	S	0	S	0	0	0	0
miR-873-5p	**S, L**	0	**S, L**	0	S	**S, L**	0	0	0	0	0

All miRNA-lncRNA interactions were validated with LncBase database. In the table, S: Starbase database, L: LncBase database. “0” denotes there is no interaction between the intended miRNA and lncRNA. Edges that passed the validation process has been bolded.

### Protein-miRNA-long non-coding RNA tripartite network

**FIGURE 4:**
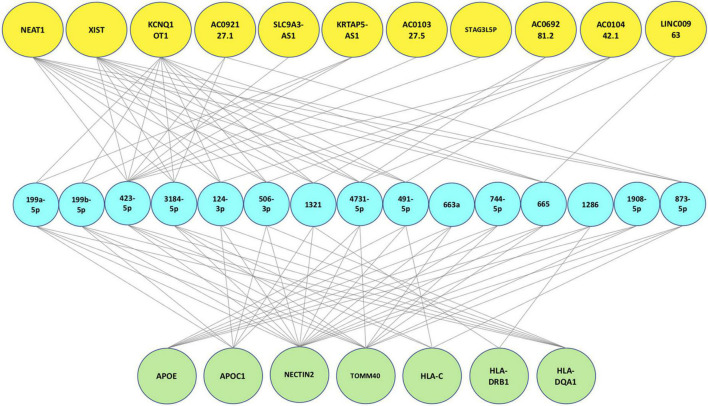
Final protein-miRNA-lncRNA tripartite network. The first layer (green nodes) consists of 7 proteins. The second layer (blue nodes) consists of 15 high degree miRNAs and the last layer (yellow nodes) consists of 11 high degree lncRNAs.

After the validation process, the final tripartite network was constructed. This network consists of 7 proteins achieved from mapping signatures to the reference genome, 15 high-degree miRNAs with 49 interactions retrieved from the protein-miRNA bipartite network and 11 high-degree lncRNAs with 45 interactions derived from the miRNA-lncRNA bipartite network. Figure 4 shows the final network.

### Scoring function results

**FIGURE 5:**
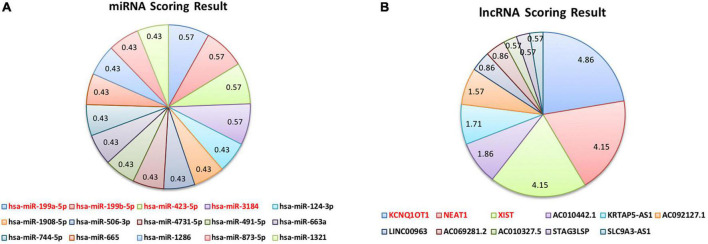
Scoring function results.

We assigned a score to each ncRNA based on the scoring function and arranged them in descending order. According to Figure 5 and Table 6, the miRNAs with the highest scores are hsa-miR-199a-5p, hsa-miR-199b-5p, and hsa-miR-3184. Moreover, KCNQ1OT1, NEAT1, and XIST are the most significant lncRNAs. Each of the mentioned ncRNAs has a score higher than the average. A literature review also confirms the significance of the discussed ncRNAs.

**TABLE 6:** The score of miRNAs and lncRNA.

miRNA	# of proteins	lncRNA	# of miRNAs
**hsa-miR-199a-5p**	4	**KCNQ1OT1**	10
**hsa-miR-199b-5p**	4	**NEAT1**	9
**hsa-miR-423-5p**	4	**XIST**	9
**hsa-miR-3184**	4	AC010442.1	4
hsa-miR-124-3p	3	KRTAP5-AS1	3
hsa-miR-1908-5p	3	AC092127.1	3
hsa-miR-506-3p	3	LINC00963	2
hsa-miR-4731-5p	3	AC069281.2	2
hsa-miR-491-5p	3	AC010327.5	1
hsa-miR-663a	3	STAG3LSP	1
hsa-miR-744-5p	3	SLC9A3-AS1	1
hsa-miR-665	3		
hsa-miR-1286	3		
hsa-miR-873-5p	3		
hsa-miR-1321	3		

Boded miRNAs and lncRNAs are more important since their scores are greater than the average score.

### MicroRNA pathway enrichment analysis

The top 10 high-degree miRNAs from the protein-miRNA bipartite network were applied to pathway enrichment analysis using the miRpathDB database (Vlachos et al., 2015). Table 7 demonstrates that the genes involved in Alzheimer’s disease pathway, Type II diabetes pathway, Insulin signaling pathway, MAPK signaling pathway, PI3K-Akt signaling pathway, mTOR signaling pathway, and Neurotrophin signaling pathway are regulated by the significant miRNAs identified in this study. The mentioned pathways have also been identified as shared pathways between DM and AD in previous research.

**TABLE 7:** MiRNA pathway enrichment analysis.

miRNA	Pathway	Database	P-value	Method
hsa-miR-199a-5p	Alzheimer’s disease	KEGG	0.04	Experimental
	Type II diabetes mellitus	KEGG	0.02	Experimental
	Insulin signaling pathway	KEGG	0.03	Experimental
	mTOR signaling pathway	KEGG	0.04	Experimental
	MAPK signaling pathway	KEGG	0.03	Experimental
	Neurotrophin signaling pathway	KEGG	0.01	Experimental
	Neuronal System	Reactome	0.02	Experimental
hsa-miR-199b-5p	Diseases of signal transduction	Reactome	0.03	Experimental
	Regulation of insulin receptor	GO-Biological Process	0.02	Experimental
	Regulation of oxidative stress	GO-Biological Process	0.03	Experimental
	Cognition	GO-Biological Process	0.03	Experimental
	Neuron differentiation	GO-Biological Process	0.04	Experimental
	Insulin signaling pathway	KEGG	0.02	Predicted
hsa-miR-423-5p	mTOR signaling pathway	KEGG	0.02	Predicted
	MAPK signaling pathway	KEGG	0.03	Predicted
	Cell cycle	KEGG	0.01	Experimental
	Oxidative stress	Reactome	0.04	Experimental
	Synapse	GO-Cellular Component	0.01	Experimental
	Regulation of cell death	GO-Biological Process	0.03	Experimental
	mTOR signaling pathway	KEGG	0.01	Predicted
	Neurotrophin signaling pathway	KEGG	0.01	Predicted
hsa-miR-3184	Alzheimer’s disease	WikiPathways	0.04	Predicted
	Insulin signaling pathway	WikiPathways	0.02	Predicted
	mTOR signaling pathway	KEGG	0.01	Predicted
hsa-miR-124-3p	Neurotrophin signaling pathway	KEGG	0.04	Experimental
	PI3K-Akt signaling pathway	KEGG	0.02	Experimental
	Lipid metabolism	Reactome	0.03	Experimental
	Insulin signaling pathway	WikiPathways	0.02	Experimental
hsa-miR-1908-5p	Aging	GO-Biological Process	0.04	Experimental
	Nervous system development	GO-Biological Process	0.04	Experimental
	Type II diabetes mellitus	KEGG	0.009	Predicted
	Insulin signaling pathway	WikiPathways	0.04	Predicted
hsa-miR-506-3p	Generation of neurons	GO-Biological Process	0.02	Experimental
	Insulin signaling pathway	WikiPathways	0.01	Predicted
	MAPK signaling pathway	KEGG	0.01	Predicted
hsa-miR-4731-5p	Neuronal System	Reactome	0.00005	Predicted
	Oxidative stress	Reactome	0.002	Predicted
	MAPK signaling pathway	KEGG	0.02	Predicted
	Insulin signaling pathway	KEGG	0.007	Predicted
hsa-miR-491-5p	PI3K-Akt signaling pathway	KEGG	0.02	Experimental
	Apoptosis	KEGG	0.03	Experimental
	AGE/RAGE pathway	WikiPathways	0.03	Experimental
	Oxidative stress	Reactome	0.04	Experimental
hsa-miR-663a	MAPK signaling pathway	KEGG	0.01	Experimental
	PI3K-Akt signaling pathway	KEGG	0.02	Experimental
	Apoptosis	KEGG	0.04	Experimental
	Aging	GO-Biological Process	0.008	Experimental
	mTOR signaling pathway	KEGG	0.04	Predicted
	Insulin signaling pathway	KEGG	0.04	Predicted
hsa-miR-744-5p	mTOR signaling pathway	KEGG	0.005	Experimental
	Insulin signaling pathway	KEGG	0.0006	Experimental
	Neurotrophin signaling pathway	KEGG	0.02	Experimental
hsa-miR-665	Neuronal System	Reactome	0.01	Predicted
hsa-miR-1286	mTOR signaling pathway	KEGG	0.04	Predicted
	Type II diabetes mellitus	KEGG	0.03	Predicted
	MAPK signaling pathway	KEGG	0.006	Predicted
	Neurotrophin signaling pathway	KEGG	0.03	Predicted
hsa-miR-873-5p	MAPK signaling pathway	KEGG	0.02	Predicted
	mTOR signaling pathway	KEGG	0.02	Predicted
	Neurotrophin signaling pathway	KEGG	0.02	Predicted
hsa-miR-1321	Insulin signaling pathway	KEGG	0.0008	Predicted
	Neurotrophin signaling pathway	KEGG	0.04	Predicted
	mTOR signaling pathway	KEGG	0.02	Predicted

## Discussion

Using a variety of data (SNPs, proteins, miRNAs, and lncRNAs), this study investigated the relationship between DM and AD and introduced candidate biomarkers that could be identified as potential drug targets for the prevention and treatment of both diseases. In addition, the current study demonstrated that the proposed candidate proteins could involve known biomarkers in the cross-talk between DM and AD, confirming the accuracy of the final tripartite network, which was supported by the literature. Moreover, many of the proposed ncRNAs have been identified in both diseases or in at least one of them.

Apolipoprotein E, the brain cholesterol transporter, appears to be highly expressed in the brain, liver, kidney, and adipose tissue and plays a crucial role in lipid metabolism. It is located on chromosome 19, and its allele number 4 (APOE4) is associated with AD. Previous research has demonstrated that this protein plays an important role in the progression of related diseases (Peila et al., 2002; Irie et al., 2008; Shinohara et al., 2020). Studies indicate that APOE dysregulation can disrupt insulin signaling by preventing it from interacting with its receptor, as well as affecting the clearance of amyloid beta (Aβ) plaques in the brain. In addition, its negative correlation with IDE brain expression may have an effect on Aβ plaques (Du et al., 2009). Apolipoprotein C1 (APOC1), the downstream gene of APOE, is involved in lipid metabolism and is predominantly expressed in the brain and liver. Patients with T2DM have a high concentration of this protein, which causes hyperlipidemia and, consequently, insulin resistance (Bouillet et al., 2016). APOC1 dysregulation has also been detected in mice with memory loss (Abildayeva et al., 2008).

In a previous GWAS study, specific alleles of “Translocase of Outer Mitochondrial Membrane 40” (TOMM40) and “Nectin cell adhesion molecule 2” (NECTIN2; i.e., PVRL2) were found to be associated with an increased risk of developing both DM and AD (Hao et al., 2015). TOMM40, an APOE-neighboring gene, is involved in age-related neurodegeneration processes (Gottschalk et al., 2014). Dysregulation of this protein induces mitochondrial dysfunction, which interferes with insulin uptake in the brain, resulting in insulin resistance (Wennberg et al., 2016). In addition, it has been reported that the expression of TOMM40 is reduced in the blood of Alzheimer’s patients (Lee et al., 2012). NECTIN2, a gene close to APOE, is a significant gene in AD progression (Hu et al., 2019). It is found in the brain and neuronal cells and is necessary for proper cell junction formation. The expression of this protein was found to be lower in T2DM patients (Kleinstein et al., 2019).

In addition to the previously mentioned proteins, the major histocompatibility complex, class I, C (HLA-C), the major histocompatibility complex, class II, DQ alpha 1 (HLA-DQA1), and the major histocompatibility complex, class II, DR beta 1 (HLA-DRB1) have been linked to AD, Parkinson’s disease, and multiple sclerosis. Additionally, their connection to DM has been documented. Studies confirmed the role of HLA-DRB1 in the progression of AD (Lu et al., 2017) and T2DM (Williams et al., 2011). Furthermore, the dysregulation of HLA-DQA1 in AD (Kwok et al., 2018) and T2DM has been discussed (Ma et al., 2013). Russell et al. (2019) also demonstrated the upregulation of HLA-C in Type 1 diabetes.

Hsa-miR-199a-5p, one of the miRNAs with the highest score as determined by the scoring function (see Table 6), has been cited in multiple studies as the dysregulated miRNA in DM and AD. It appears to be expressed in the brain and controls GLUT4, the brain glucose transporter. GLUT4 is heavily expressed in the hippocampus and is extremely important for hippocampal memory function (McNay and Pearson-Leary, 2020). It is upregulated in the prefrontal cortex of Alzheimer’s patients (Heidari et al., 2018) as well as diabetic patients’ plasma (Yan et al., 2014) which disrupts the regulation of GLUT4, preventing neurons from absorbing insulin and causing insulin resistance. An additional target of hsa-miR-199a-5p in the brain peptidylprolyl *cis*/*trans* isomerase, NIMA-interacting 1 (PIN1), an enzyme that regulates protein function in the post phosphorylation process. It is believed that the lack of PIN1 (downregulation) causes tau hyperphosphorylation in the brain (Sultana et al., 2006). Hsa-mir-199b-5p also interacts with PIN1. It is upregulated in the brains of Alzheimer’s patients, and causes hyperphosphorylation of tau protein (Heidari et al., 2018).

Hsa-mir-124-3p has been downregulated in DM and AD. One of the most important targets of this miRNA is Beta-secretase 1 (BACE1) which is a significant known biomarker for AD. Decreasing the expression of hsa-mir-124-3p causes increase in the levels of BACE1, leading to hyperphosphorylation of the tau protein (Fang et al., 2012; Lau et al., 2013).

Hsa-miR-1908-5p, which interacts with APOE in the tripartite network, is involved in the regulation of human obesity and cholesterol. Wang et al. demonstrated that upregulation of this miRNA in the peripheral blood cells of Alzheimer’s patients could inhibit Aβ clearance (Wang et al., 2019). In addition, the role of this microRNA in cholesterol metabolism and neurodegeneration has been investigated (Goedeke and Fernández-Hernando, 2014).

Hsa-miR-423-5p has been introduced as the novel differentially expressed miRNA between non-AD individuals and Mild Cognitive Impairment (MCI) patients (Nagaraj et al., 2017). Alzheimer’s patients experience an upregulation of hsa-mir-744-5p, which may regulate PIN1 expression (Lau et al., 2014). Sidorkiewicz et al. (2020), demonstrated the role of hsa-miR-491-5p in the diagnosis of T2DM in prediabetic patients. They discussed the upregulation of this miRNA as the diagnostic biomarker for T2DM (Sidorkiewicz et al., 2020). Downregulation of hsa-miR-665 has also been identified in T2DM patients (Yang et al., 2017). Additionally, Sheinerman et al. (2012) presented this miRNA as a novel plasma biomarker for the diagnosis of MCI.

Alzheimer’s patients have been found to have altered expression of hsa-miR-1321 (Lau et al., 2014), hsa-mir-506-3p (Nunez-Iglesias et al., 2010), hsa-miR-663a (Grasso et al., 2019), and hsa-miR-873-5p (Shi et al., 2018). These miRNAs, along with hsa-miR-4731-5p, hsa-miR-1286, and hsa-miR-3184-5p, have been introduced in this study as novel candidate miRNAs in the cross-talk between DM and AD for further analysis. The miRNAs identified in this study were validated using the Human microRNA Disease Database (2007). Results show that hsa-miR-124-3p is a significant miRNA in T2DM and AD. In addition, hsa-mir-665 and hsa-mir-199a-5p have been identified as T2DM biomarkers, whereas hsa-miR-199b-5p and hsa-miR-744-5p have been discovered in inflammatory and brain diseases, respectively. As a plasma biomarker for MCI and glioblastoma, the function of hsa-miR-491-5p has been discussed.

According to Table 6, KCNQ1OT1 is the highest score lncRNA that is highly expressed in the brain. In addition, the interaction between KCNQ1OT1 and hsa-miR-506 has been identified in the plasma of patients with elevated glucose levels (Zhu et al., 2020).

Alzheimer’s patients have upregulated levels of NEAT1 and XIST. They may result in the downregulation of hsa-miR-124-3p, which in turn causes the upregulation of BACE1. As previously stated, BACE1 dysregulation results in tau hyperphosphorylation, which ultimately leads to cell death. Therefore, silencing the aforementioned lncRNAs may reduce AD-related complications (Zhao et al., 2019; Huang et al., 2020; Yue et al., 2020). In addition, NEAT1 was found to be significantly upregulated in diabetic rats, leading to a highly activated Akt/mTOR signaling pathway (Huang et al., 2019). The role of XIST in insulin resistance and T2DM has also been reported (Sathishkumar et al., 2018).

Long intergenic non-coding RNA 963 (LINC00963) also modulates the Foxo signaling pathway in rats and induces oxidative stress (Chen et al., 2018).

The role of other achieved lncRNAs has been less proven. AC011481.2, TSBP1, and BTNL2 Antisense RNA 1 (TSBP1-AS1) were directly obtained by mapping signatures to the reference genome (see Table 2). AC011481.2 is a novel antisense transcript for NECTIN2. The GWAS catalog identifies mutations in specific loci (rs6857) of this gene as a risk factor for developing T2DM and AD simultaneously (2008). This mutation has been identified as one of 127 common SNPs in the cross-talk between DM and AD (see Supplementary Table 1). TSBP1-AS, KRTAP5-1/KRTAP5-2 antisense RNA 1 (KRTAP5-AS1), STAG3LSP, SLC9A3 antisense RNA 1 (SLC9A3-AS1), AC092127.1, AC010327.5, AC069281.2, and AC010442.1 have an unknown function in DM and AD. Lack of information about lncRNAs may be the reason. Due to the fact that they have been identified as significant in our study, additional analysis may lead to noteworthy advancements. Table 8 provides a summary of the discussion.

**TABLE 8:** Summary of discussion.

Candidate biomarker	Significant target genes	Description
hsa-miR-199a-5p	GLUT4, PIN1	Upregulated in DM and AD, causes the downregulation of target genes, resulted in hyperphosphorylation of Tau and cell death.
hsa-miR-199b-5p	PIN1	Upregulated in DM and AD, causes downregulation of PIN1, resulted in hyperphosphorylation of Tau.
hsa-miR-124-3p	BACE1, APOC1	Downregulated in DM and AD, causes upregulation of target genes, resulted in hyperphosphorylation of Tau and changing in lipid metabolism.
hsa-miR-1908-5p, hsa-miR-491-5p	APOE	Upregulated in DM and AD, causes downregulation of APOE, resulted in failing the clearance of Aβ plaques.
hsa-miR-423-5p	NECTIN2, TOMM40, HLA-C, HLA-DQA1	Dysregulated in AD causes changing in the regulation of target genes.
hsa-miR-744-5p	APOE, PIN1	Upregulated in AD, causes downregulation of target genes, resulted in failing the clearance of Aβ plaques and hyperphosphorylation of Tau.
hsa-miR-665	APOE	Downregulated in DM and AD, causes upregulation of APOE, resulted in changing in lipid metabolism.
hsa-miR-506-3p, hsa-miR-663a, hsa-miR-873- 5p, hsa-miR-1321	APOE, APOC1, TOMM40, NECTIN2, HLA-C, HLA-DQA1, HLA-DRB1	Dysregulated in AD. Introduced in this study as novel miRNAs for further analysis.
hsa-miR-4731-5p, hsa-miR-1286, hsa-miR-3184-5p	APOC1, TOMM40, NECTIN2, HLA-C, HLA-DQA1, HLA-DRB1	Introduced in this study as novel miRNAs for further analysis.
NEAT1, XIST	has-miR-124-3p, BACE1	Upregulated in AD and DM, causes downregulation of hsa-miR-124-3p, resulted in upregulation of BACE1 and hyperphosphorylation of Tau.
KCNQ1OT1	hsa-miR-199a-5p, hsa-miR-199b-5p, hsa-miR-124-3p	Dysregulated of this lncRNAs could lead to oxidative stress.
LINC00963	has-miR-665	Causes oxidative stress by target Foxo signaling pathway.
AC011481.2		Increased risk of developing T2DM and AD simultaneously by mutation in a specific loci (rs6857).
KRTAP5-AS1, STAG3LSP, SLC9A3-AS1, AC092127.1, AC010327.5, AC069281.2	hsa-miR-199a-5p, hsa-miR-199b-5p, hsa-423-5p	Introduced in this study as novel lncRNAs in the cross-talk between DM and AD.

In future research, we will increase the reliability of our study by evaluating the biomarkers we have introduced. In this regard, at the Royan Institute for Stem Cells, we intend to conduct a mouse-based laboratory experiment.

## Conclusion

We identified 127 shared SNPs, 7 proteins, 15 miRNAs, and 11 lncRNAs in the cross-talk between DM and AD. According to the literature review, the proteins APOE, APOC1, TOMM40, NECTIN2, HLA-C, HLA-DRB1, and HLA-DQA1, discovered in this study, play crucial roles in the progression of both or one of the aforementioned diseases. Furthermore, the scoring function revealed that hsa-miR-199a-5p, hsa-miR-199b-5p, hsa-miR-423-5p, and hsa-miR-3184-5p (miRNAs with a score greater than 0.5) are the most significant miRNAs discovered in this study; the first two are known miRNAs in the progression of corresponding diseases, and the last two are proposed. This study also discovered that NEAT1, XIST, and KCNQ1OT1 (lncRNAs with above-average scores) are involved in the cross-talk between DM and AD. The first two lncRNAs are known to play a role in the progression of disease (particularly AD), and the third has been identified as a candidate for future research. Finally, we proposed lncRNA/miRNA pairs; hsa-miR-124-3p/KCNQ1OT1, hsa-miR-124-3p/NEAT1, and hsa-miR-124-3p/XIST, all of which are expressed in the brain, as new candidate biomarkers for the occurrence of DM and AD simultaneously. In addition, dysregulation in the expression of hsa-miR-199a-5p in the blood of patients has been suggested for early detection of both diseases.

## Data availability statement

The original contributions presented in this study are included in the article/Supplementary material, further inquiries can be directed to the corresponding authors.

## Author contributions

ShG designed and performed experiments, analyzed data, and wrote the manuscript. CE, SaG, KS, and MH-R supervised the research. All authors read and approved the final manuscript.

## Footnotes

1
http://www.nealelab.is/uk-biobank

2
https://ldlink.nci.nih.gov

3
https://genome.ucsc.edu/

4
https://www.snp-nexus.org/v4/
